# RETRACTION: Effect of quality nursing intervention on wound healing in patients with burns: A meta‐analysis

**DOI:** 10.1111/iwj.70079

**Published:** 2024-09-24

## Abstract

Retraction: LiuL. , LiuZ.‐F. , ZhuH.‐Y. , XuH.‐Q. , “Effect of Quality Nursing Intervention on Wound Healing in Patients with Burns: A Meta‐Analysis,” International Wound Journal 21, no. 3 (2023): e14717, 10.1111/iwj.14717.PMC1091237538439182

The above article, published online on 03 March 2024, in Wiley Online Library (http://onlinelibrary.wiley.com/), has been retracted by agreement between the journal Editor in Chief, Professor Keith Harding; and John Wiley & Sons, Ltd. It came to the publisher's attention from a third party that a number of articles shared concerning similarities in format and structure. Following an investigation by the publisher, the retraction has been agreed on as the peer review and publishing process for this article were found to be manipulated.

Retraction: L. Liu , Z.‐F. Liu , H.‐Y. Zhu , H.‐Q. Xu , “Effect of Quality Nursing Intervention on Wound Healing in Patients with Burns: A Meta‐Analysis,” International Wound Journal 21, no. 3 (2023): e14717, 10.1111/iwj.14717.PMC1091237538439182

The above article, published online on 03 March 2024, in Wiley Online Library (http://onlinelibrary.wiley.com/), has been retracted by agreement between the journal Editor in Chief, Professor Keith Harding; and John Wiley & Sons, Ltd. It came to the publisher's attention from a third party that a number of articles shared concerning similarities in format and structure. Following an investigation by the publisher, the retraction has been agreed on as the peer review and publishing process for this article were found to be manipulated.

